# Birdsong as an Indicator of Habitat Structure and Quality

**DOI:** 10.1002/ece3.71510

**Published:** 2025-06-04

**Authors:** Katalin Krenhardt, Mónika Jablonszky, Karola Anna Barta, Miklós Laczi, Gergely Nagy, Sándor Zsebők, László Zsolt Garamszegi

**Affiliations:** ^1^ Evolutionary Ecology Research Group, Institute of Ecology and Botany HUN‐REN Centre for Ecological Research Vácrátót Hungary; ^2^ Behavioural Ecology Group, Department of Systematic Zoology and Ecology ELTE Eötvös Loránd University Budapest Hungary; ^3^ HUN‐REN–PE Evolutionary Ecology Research Group University of Pannonia Veszprém Hungary; ^4^ Doctoral School of Biology, Institute of Biology ELTE Eötvös Loránd University Budapest Hungary; ^5^ HUN‐REN–ELTE–MTM Integrative Ecology Research Group ELTE Eötvös Loránd University Budapest Hungary

**Keywords:** acoustic adaptation, acoustic communication, collared flycatcher, habitat quality, vegetation structure

## Abstract

Birdsong is a complex and highly flexible sexual signal that plays a crucial role in intra‐ and intersexual communication. Various aspects of the surrounding habitat can influence birdsong; for example, birds may modify their songs to enhance acoustic transmission, or males in high‐quality territories with abundant food resources may produce more elaborated songs. However, these mechanisms remain largely unexplored in many natural systems. In our field study, we recorded the songs of male‐collared flycatchers ( *Ficedula albicollis* ) in a Hungarian population, alongside detailed habitat variables reflecting structure and quality. We analysed song traits describing frequency, temporal structure and complexity. Habitat variables included canopy closure, Shannon diversity index of the tree species, mean trunk circumference of dominant tree species and tree health status. We revealed that mean song frequency was negatively associated with canopy closure. This result likely reflects birds adjusting the frequency of their songs to the acoustic properties of their environment, or it may be explained by quality‐dependent territory selection or the use of high‐performance songs to signal territory quality. Our results have implications for the study of sexual selection and how birds adapt to different environments and suggest that aspects of birdsong can reflect habitat quality.

## Graphical abstract

We investigated the relationship between song traits of the collared flycatcher ( *Ficedula albicollis* ) and habitat structure and quality. We found that collared flycatcher males sing at lower frequencies when the canopy is more closed, which may be an adaptive plastic response to the acoustic properties of their environment, or in this way they can signal the quality of their territories.

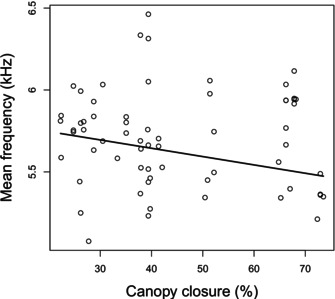

## 1 Introduction

Birdsong serves as a multifaceted trait in avian communication and plays a substantial role in sexual selection. The elaborate vocalisations of male songbirds (as well as other animals) play a role in establishing and defending territories, signalling their presence to rivals while simultaneously attracting potential mates (Eriksson and Wallin 1986; Lundberg and Alatalo 1992; Catchpole and Slater 2008). This complex and highly variable behavioural trait could convey essential information about the signaller's identity, quality and occupied territory to conspecifics, facilitating territory defence and mate selection (Qvarnström et al. 2010; Vehrencamp et al. 2014; Warrington et al. 2014). By relying on long‐range acoustic signals, animals resolve conflicts over resources, such as territory and/or potential mates, without engaging in resource‐intensive physical confrontations, thus optimising energy allocation and minimising the risks associated with direct encounters (Peake et al. 2001; Searcy and Beecher 2009; Maynard et al. 2012). Acoustic communication, therefore, plays a pivotal role in the social life of individuals, including reproductive interactions, enabling them to convey messages efficiently and adaptively in their natural environments (Brumm and Naguib 2009).

The remarkable plasticity observed in birdsong at the within‐species level underscores its dynamic nature, influenced by an intricate interplay of individual‐specific and environmental factors (Gil and Gahr 2002; Arnold et al. 2010). This plasticity allows birds to adjust their song characteristics, such as frequency, complexity, repertoire size and song length, to various internal and external factors (Garamszegi, Møller, et al. 2004; Motes‐Rodrigo et al. 2017; Zipple et al. 2019). Internal factors, such as immune state (Garamszegi, Møller, et al. 2004), age (Zipple et al. 2019), reproductive status (Warrington et al. 2014) and breeding experience (Motes‐Rodrigo et al. 2017) may exert significant effects on the within‐individual variation in song characteristics. Song characteristics are also known to vary with various external factors, such as population and social dynamics (Geberzahn and Aubin 2014; Gersick and White 2018; Henderson et al. 2018), habitat quality (Hoi‐Leitner et al. 1995; Bueno‐Enciso et al. 2016; Zsebők et al. 2017), predation risk (Schmidt and Belinsky 2013) or even temperature (Strauß et al. 2020).

The plasticity of birdsong would be particularly advantageous in response to varying habitat characteristics, as habitat quality exerts profound effects on birdsong transmission and reception within forest environments (Brumm and Naguib 2009). For example, in dense, closed habitats, such as tropical rainforests, sound waves experience increased scattering and consequent attenuation due to the complex vegetation structure that can affect higher frequencies (Wiley and Richards 1982). Scattering and reverberation caused by the foliage, trunks and branches can result in reduced signal clarity and effective communication range, posing challenges for birds attempting to convey information through vocalisations and favouring slower and lower frequency songs (Wiley and Richards 1982; Naguib 2003). Conversely, in more open habitats, such as savannahs or grasslands, where vegetation is sparse and spaced out, sound transmission may be less obstructed, allowing for clearer and more long‐range communication (Naguib 2003; Boncoraglio and Saino 2007). However, strong wind can pose difficulties for communication here, attenuating especially higher frequency sounds (Morton 1975). Additionally, variations in foliage type, such as deciduous versus coniferous trees, can also impact the acoustic properties of the environment, further influencing birdsong characteristics (Blumenrath and Dabelsteen 2004; Attenborough and Taherzadeh 2016), such as mean frequency and frequency bandwidth. Furthermore, the spatial arrangement and width of trees within a habitat can create acoustic microhabitats, with areas of high sound reflection or absorption, affecting the perceived quality of birdsong signals (Bradbury and Vehrencamp 2011). There is evidence from various bird species that individuals can adjust their songs to maximise sound transmission altered by these habitat characteristics using low frequencies and narrow frequency bandwidth in dense habitats (Nicholls and Goldizen 2006; Benedict and Warning 2017; Sebastián‐González et al. 2018), as stated by the acoustic adaptation hypothesis (Morton 1975). Thus, the intricate relationship between habitat structure and birdsong characteristics underscores the importance of considering environmental factors in understanding avian communication strategies.

Beyond its direct impact on birdsong transmission and reception, habitat quality exerts indirect effects on avian vocalisations through its influence on resource availability and distribution (Traba et al. 2022). Variations in habitat structure, such as vegetation composition and spatial arrangement, can significantly alter the abundance and accessibility of resources critical for avian survival and reproduction, consequently shaping birdsong characteristics (Alatalo et al. 1990; Martinez‐Almoyna et al. 2024). For instance, studies have demonstrated that habitats with greater vegetation density, cover and heterogeneity attract higher densities of insect prey, a vital food source for many insectivorous bird species (Prather and Kaspari 2019; Traba et al. 2022; Martinez‐Almoyna et al. 2024). In turn, the availability of abundant food resources in these habitats may enhance the physical condition and nutritional status of resident birds, potentially influencing performance‐related acoustic traits, such as song rate or song length (Strain and Mumme 1988; Alatalo et al. 1990; Yamada and Soma 2016). Additionally, habitat quality can affect the distribution of suitable nesting sites and breeding territories, further impacting social dynamics and mate selection processes within avian communities. Although these factors are expected to trigger also within‐individual changes in song, due to the difficulty of recording the same birds at multiple territories under natural conditions, their effects were investigated unfortunately only at the among‐individual level (Hoi‐Leitner et al. 1995; Goretskaia et al. 2018; Garcia et al. 2023). Birds occupying high‐quality nesting habitats often exhibit more elaborate and structurally complex songs or higher song rates compared to individuals in suboptimal habitats, possibly as a signal of territory quality and mate attractiveness (Hoi‐Leitner et al. 1995; Goretskaia et al. 2018; Garcia et al. 2023). Thus, by indirectly influencing resource availability and breeding success, habitat quality plays a pivotal role in shaping the acoustic signals and behavioural strategies employed by birds in their natural environments.

While numerous studies have explored the connections between habitat structure and bird song in various species (see the meta‐analysis of Boncoraglio and Saino 2007), there is a lack of studies investigating this relationship treating song as an individual‐specific trait in species with complex and variable song and that simultaneously consider multiple acoustic and habitat characteristics. Therefore, this study aims to elucidate the relationships between male song quality and the habitat structure of their territories in a population of free‐living collared flycatchers ( *Ficedula albicollis* ). Collared flycatchers have complex songs, as males have 30 syllable types on average that can be flexibly arranged into songs (Garamszegi et al. 2006). Variations in habitat structure, such as variation in forest canopy closure, trunk size of the dominant tree species and shrub cover were associated with the acoustic properties of the environment, as sounds scatter on these surfaces and become attenuated in a frequency‐dependent manner (Wiley and Richards 1982). Thus, we predicted that areas with greater canopy closure, wider tree trunks and denser shrub layers would be associated with singing at lower mean frequencies and with narrower frequency bandwidths, as increased levels of signal attenuation at higher frequencies make this advantageous. Denser foliage that harbours more caterpillars (Marshall and Cooper 2004), the main food resources of flycatchers and tits during chick‐rearing (Török 1986; Charmantier et al. 2008; Samplonius et al. 2016), could indicate better quality territories and would influence the performance‐related song traits and song complexity. Accordingly, we predicted that males occupying high‐quality habitats, including high tree species diversity, more closed canopy and healthier trees, would produce more complex and longer songs and greater repertoire sizes compared to those in lower‐quality habitats. By testing these hypotheses, we could contribute to a deeper understanding of how environmental factors shape avian communication strategies, shedding light on the potential role of birdsong in dynamically signalling natural landscapes.

## 2 Materials and Methods

### 2.1 Study Site and Model Species

Data were obtained from a population of collared flycatchers breeding in the Pilis‐Visegrádi Mountains (47°43′N, 19°01′ E), Hungary. The study population is located in a continuous, mainly oak‐dominated woodland, with the sessile oak ( *Quercus petraea* ) as the dominant tree species. The forest is more or less homogeneous, except for some dirt roads and clearings. The study site is within the protected area of the Duna‐Ipoly National Park and the Pilisi Parkerdő Zrt and is practically unmanaged, with dead trees left in place. The area contained approximately 600 nest boxes on average at 31 m from each other during the study period, mainly occupied by collared flycatchers. The cavity of the next boxes is 24 × 11 × 11 cm and the diameter of the entrance hole is 3.2 cm (Lambrechts et al. 2010).

The collared flycatcher is a small, insectivorous, relatively short‐lived (2.46 years on average for males based on our long‐term ringing records), long‐distance migratory bird that breeds mainly in deciduous forests, including those in Central Europe (Cramp 1993). The majority of the birds are socially monogamous, although polygyny occurs regularly and is around 6% in our study area (Garamszegi, Török, et al. 2004). Only the females build the nest and incubate the eggs, but males provide parental care during chick‐feeding. After spring migration from their winter quarters in Africa, males arrive earlier (around the middle of April) to the breeding grounds than females. After arrival, males establish their small territories around their chosen nest boxes, then they begin singing.

The primary function of the song performance in collared flycatcher males is the dual purpose of attracting potential mates and dissuading rivals. The song performance comprises sequences of songs, each separated by brief intervals (i.e., by a few seconds). Typically, songs last 3–5 s, and they consist of 2–20 distinct syllables (Gelter 1987). Collared flycatcher males exhibit a moderate repertoire size, estimated to range between 20 and 100 syllable types, derived from an analysis of 20 songs per individual (Garamszegi et al. 2006; Zsebők, Herczeg, et al. 2018). We do not know much about song learning in the study species, but it seems to be an open‐ended learner (Garamszegi et al. 2007; Zsebők et al. 2021; Vaskuti et al. 2022), similarly to its sister species, the pied flycatcher ( *Ficedula hypoleuca* ) (Eriksen et al. 2011). The characteristics of the song are linked to various aspects of individual quality, including age and immune state (Garamszegi, Møller, et al. 2004; Garamszegi et al. 2006, 2007) and are closely associated with mating success and male–male competition dynamics (Garamszegi, Møller, et al. 2004; Hegyi, Szöllősi, et al. 2010). Moreover, the low repeatability estimates of song traits found in the study species imply that these traits have considerable within‐individual variance, even over short time periods (Zsebők et al. 2017), enabling their habitat‐dependent alteration.

### 2.2 Song Recordings

We obtained 94 recordings from 50 collared flycatcher males (we had more than one recordings from 24 males) during the three courtship seasons between 2020 and 2022. The recordings were made between April 19 and May 3, and from 8:37 to 14:07.

We monitored the study area daily for newly arrived, unpaired birds in the morning, as it is the most active singing period of the day. The sound of the focal males was recorded by a Telinga parabola dish with Sennheiser ME62 microphone and K6 preamplifier on Zoom H4n digital recorders (with 44.1 kHz sampling rate and 16 bit quality). The recordings lasted at least 10 min and contained at least 20 good quality songs. We made sound recordings only in relatively good weather conditions without rain and strong wind. As part of another study, we presented the focal male a live female decoy for 5–10 min in certain cases, and we recorded its song after removing the stimulus bird (the procedure and results are described in detail elsewhere, Jablonszky et al. 2021; Jablonszky et al. 2022). We made 51 recordings with a live female decoy on the focal male's territory and 43 recordings without a decoy, and we controlled for these methodological differences in our statistical analyses. All repeated recordings were made on the same territory as the first one; the males did not change territories.

After performing song recordings, we captured the males within an hour by using spring traps (two metal sheets with a spring between them) in their nest boxes for ringing. Based on our decades‐long field experience, males do not switch territories in such a short time, so we are highly confident that we captured the male that had been recorded at the same nest box. We marked the males with individually numbered rings (Aranea, Poland; standard rings of the Hungarian Bird Ringing Centre) for long‐term identification (if they did not already have one), following the standard ringing protocol. We determined the age of males based on their feather colouration (Svensson 1992), as one‐year‐old flycatcher males bear brown remiges and smaller white wing patches, while the remiges of older males are black and they have larger wing patches. Regarding the age of the males, there were 14 one‐year‐old, 29 more than one‐year old and 7 males with age unknown (not caught) in our analyses. We have included males of unknown age in the analyses only for specific song traits not related to age. We also marked the males individually with water‐resistant pens using colour combinations, allowing us to identify and record them repeatedly in the following days without the need of recapturing them again (reading the rings would have required recapture).

### 2.3 Ethical Notes

All applicable institutional, national and/or international guidelines for the care and use of animals were followed. Permissions for the fieldwork have been provided by the Middle‐Danube‐Valley Inspectorate for Environmental Protection, Nature Conservation and Water Management (reference numbers: PE/EA/101‐8/2018, PE‐06/KTF/8550‐4/2018 and PE‐06/KTF/8550‐5/2018) and were approved by the ethical committee of the Eötvös Loránd University (reference number: TTK/2203/3).

Song recording is a non‐invasive method, and none of the males were harmed during capture. Female decoys were presented to the focal males in small cages (15 × 20 × 15 cm) during the song recordings, which protected the decoys from any harmful encounters with the focal males or other females in the surroundings. After the song recordings, we placed the decoys in larger cages (40 × 24 × 40 cm) covered with sheets to minimise the level of stress, and provided them food (i.e., mealworms) and fresh water ad libitum. The decoys were held in captivity for as few days as possible, and they were released at the site of capture after we made sure they were in good condition (i.e., proper body mass, active and vivid behaviour). It was shown before that the time spent in captivity did not have any long‐term effects on the decoys, as their reproductive success and survival in the given year did not differ from those of other flycatchers in the population (Garamszegi et al. 2009).

### 2.4 Acoustic Analyses

We manually extracted songs from the recordings using Adobe Audition 3.0 software (Adobe Systems). Specifically, we selected 20 high‐quality songs per recording, where the spectrograms of syllables were clearly distinguishable from the background noise. The Ficedula Toolbox (Zsebők, Blázi, et al. 2018) was employed to determine the start and endpoints, as well as the minimum and maximum frequencies of each syllable, focusing only on dominant frequencies excluding harmonics. The time and frequency boundaries of the syllables were identified at approximately 20 dB above the background noise level, utilising a Hann FFT window with a 512‐point length and 95% window overlap in spectrographic settings.

The mean frequency of the syllables was automatically extracted by the Ficedula Toolbox from which we derived the following variables for each song: the mean frequency (average of the syllables' mean frequencies), the minimum and maximum frequencies (minimum and maximum of the syllables' mean frequencies), as well as the frequency bandwidth (maximum frequency—minimum frequency). Moreover, based on the time stamps of the syllables we calculated the song length. Additionally, we automatically clustered the syllables into syllable types. To do this, we first obtained three syllable‐level variables from the Ficedula Toolbox (duration, mean frequency, frequency bandwidth). Then, we conducted a k‐means clustering with 200 clusters on all the syllables together in the acoustic space defined by these three variables with the ‘k‐means’ function from the ‘vegan’ R package (Oksanen et al. 2016). Finally, based on the cluster identifiers of the syllables we obtained the repertoire size as the number of different syllable types in the recording and the complexity for each song defined by the number of different syllable types divided by the number of syllables in the songs. We then averaged the song‐level variables to obtain one value per recording. We used the recordings as the basis of further analyses, but controlled for the fact that multiple recordings originated from the same males in all models. Descriptive statistics for the song traits can be seen in Table 1.

**TABLE 1:** Pearson correlations between the investigated song traits along with their mean and standard deviation (SD).

	Mean frequency (kHz)	Frequency bandwidth (kHz)	Complexity	Song length (s)	Repertoire size
Frequency bandwidth	−0.29				
Complexity	−0.13	−0.13			
Song length	−0.14	0.56	−0.32		
Repertoire size	−0.22	0.66	−0.22	0.84	
Mean ± SD	5.65 ± 0.33	3.69 ± 0.82	0.91 ± 0.04	2.51 ± 0.81	83.30 ± 19.08

In order to assess the reliability of the clustering method, we conducted a comparison between the repertoire size estimates obtained through manual enumeration (refer to Zsebők, Blázi, et al. 2018 for detailed methodology) and those derived from the k‐means method. The correlation analysis between these two sets of estimates (Pearson's correlation, *r* = 0.81, df = 74, *p* < 10^−15^) demonstrated that the k‐means clustering method serves as a reliable substitute for the manual clustering approach (see also: Jablonszky et al. 2021).

### 2.5 Characterisation of Habitat Structure

According to a standard protocol used for vegetation assessment in deciduous forests (Ádám et al. 2013; Bereczki et al. 2014), we characterised habitat for each collared flycatcher male in a circle with a radius of 10 m drawn around the occupied nest box. With these measurements we intended to characterise the vegetation in the vicinity of the nest box from where the male was singing and not the whole acoustic space of the birds (acoustic detectability of this species is around 80 m; Poprach and Machar 2019). Within these individual territories, we described the habitat structure by five different metrics. Specifically, we characterised tree species (identified using Király 2009) diversity using the Shannon diversity index (Shannon 1948). We included trees with a circumference of more than 15 cm and a height of more than 160 cm in the calculations of the Shannon index (smaller woody plants were regarded as shrubs). We defined forest canopy closure as the percentage of the sky obscured by the trees when viewed from a single point (Jennings et al. 1999). For this, we took four photographs (with a Samsung A70 smartphone) 7 m from the nest box in each of the four cardinal directions, from a height of 1.5 m. Using ImageJ (Schneider et al. 2012), we calculated the percentage of the sky obscured by the forest canopy for each of the four photographs (Xiong et al. 2019), and then we calculated their average to describe the forest canopy closure with a single value. Furthermore, we measured the trunk circumference of each sessile oak tree (the dominant tree species in our study area) at breast height (i.e., 1.37 m above ground level) within the radius of 10 m, and we calculated their average. Moreover, we also scored each sessile oak based on its health status (i.e., 1: healthy; 2: damaged canopy, that is the presence of dry, leafless and broken branches; 3: damaged canopy and trunk, namely cracks and areas without barks on the trunk; and 4: standing dead tree higher than breast height) to calculate the proportion of healthy (i.e., score 1) sessile oaks (Ádám et al. 2013; Bereczki et al. 2014). Finally, we defined shrub cover as the percentage of the ground covered by the shrubs. For this, we estimated shrub cover in a small circle with a radius of 1 m drawn around four points which were 7 m from the nest box in each of the four cardinal directions (following protocols for similar habitats, see Ádám et al. 2013; Bereczki et al. 2014). Then, we calculated their average to describe shrub cover with a single value. Descriptive statistics for the habitat structure variables can be seen in Table 2.

**TABLE 2:** Pearson correlations between the investigated habitat variables along with their mean and standard deviation (SD).

	Canopy closure (%)	Shannon diversity index	Mean trunk circumference of sessile oaks (cm)	Health status	Shrub cover (%)
Shannon diversity index	0.23				
Mean trunk circumference of sessile oaks	−0.16	−0.23			
Health status	0.07	0.13	−0.23		
Shrub cover	0.14	0.51	−0.04	−0.16	
Mean ± SD	47.82 ± 18.51	0.65 ± 0.32	101.58 ± 12.20	0.69 ± 0.14	10.28 ± 11.02

### 2.6 Statistical Analysis

Before the analyses, we checked the correlation among the song traits (Table 1) and among the habitat structure variables (Table 2) to avoid including highly correlated variables (with similar biological meaning) in the analyses. Repertoire size was highly correlated with song length (*r* = 0.84) and frequency bandwidth (0.66), so it was excluded from further analyses. Among the habitat variables, the correlation between the Shannon diversity index and shrub cover was the highest (*r* = 0.51), so we decided to exclude shrub cover, as it was collected only in 2021 and 2022 and, consequently, had a low sample size.

We built linear mixed models (LMMs) to examine the relationship between the song variables of collared flycatcher males (mean frequency, frequency bandwidth, complexity and song length) and the habitat structure of their territories during the courtship season, while taking into account the effects of several individual‐ and context‐specific control variables. Previous studies revealed that the age of the focal male, the exact time of the song recording (i.e., year, date and hour) and the presence of a live female decoy on the focal male's territory might affect the song quality of the collared flycatcher males (Garamszegi et al. 2007; Jablonszky et al. 2021). Thus, first, we built linear mixed models to test the relationship between the song traits and control variables. Each model contained one of the song variables as the response variable, age of the individual (as a binary variable: one‐year‐old or older), year (as factor), date (days from 1st January) and time of the song recording (as continuous variables) and the presence of a female decoy (as a binary variable) as predictor variables. The random part of the models contained the individual identity of the focal male because several males had multiple song recordings. Using backward stepwise model selection, we determined the significant control variables in the case of each song variable. The random factor controlling for the independence in the data due to repeated measurements from the same individuals were always retained in the models. All continuous variables were *z*‐transformed.

Afterwards, we built linear mixed models to test the relationship between the song variables of collared flycatcher males and the habitat structure of their territories, including the retained control variables. Thus, each model contained one of the song variables as the response variable, all environmental variables characterising habitat structure and the retained control variables. The final sample sizes differed between the models due to missing values in the actual control variables included for each song trait.

We carried out rigorous model diagnostics throughout the analyses. We used Rosner's test (Rosner 1983) to identify outliers and Shapiro–Wilk test (Shapiro and Wilk 1965) to check the normal distribution of the data before model building. We found and removed three outliers in the model for frequency bandwidth, one for complexity and two for song length. After running the models but before interpreting model results, the model residuals were checked visually by inspecting scale‐location plots, histograms and q–q plots, to verify the normality, homogeneity and homoscedasticity of the residuals. Furthermore, the stability of models against influential data points, as well as absence of collinearity with Variance Inflation Factor (VIF) were verified (O'Brien 2007; Freckleton 2011). These model diagnostics verified that the data comply with the assumptions of the model.

All analyses were carried out in the R statistical environment (version: 4.3.2, R Core Team 2023). LMMs were fitted using the ‘*lmer*’ function from the ‘*lme4*’ package (Bates et al. 2015) and *p* values were calculated for these models using the *‘lmerTest’* package (Kuznetsova et al. 2017). Rosner's tests were carried out with the ‘*EnvStats*’ package (Millard 2013) and Shapiro–Wilk tests with the ‘*stats*’ package (R Core Team 2023). VIF was calculated by the ‘*vif*’ function from the ‘*car*’ package (Fox and Weisberg 2011).

## 3 Results

### 3.1 Relationships Between Song and Control Variables

Based on the results of the backward model selections for determining the most important control variables, we found that mean frequency was significantly and negatively related to the age of the focal male, as older males had lower mean frequencies and mean frequency was higher when a decoy female was displayed on the male's territory before the recording (Tables 3 and 4). We also found that frequency bandwidth was significantly and positively related to the date of the song recordings, as time passed, frequency bandwidth increased (Tables 3 and 4). Furthermore, we discovered that complexity was increasingly higher in the subsequent study years (Tables 3 and 4). Finally, we did not find any relationship between song length and any of the control variables (Table 3).

**TABLE 3:** Results from the backward model selection investigating the relationship between the song traits and the control variables.

Control variables	Mean frequency	Frequency bandwidth	Complexity	Song length
Year	*F* = 1.451, *p* = 0.245	*F* = 0.445, *p* = 0.644	***F* = 4.555**, *p* = 0.017	*F* = 0.100, *p* = 0.905
Date	*F* = 0.684, *p* = 0.412	***F* = 13.404**, *p* = 0.001	*F* = 0.009, *p* = 0.925	*F* = 1.135, *p* = 0.290
Recording time	*F* = 1.692, *p* = 0.198	*F* = 0.523, *p* = 0.472	*F* = 0.042, *p* = 0.837	*F* = 0.084, *p* = 0.772
Age	***F* = 6.003**, *p* = 0.019	*F* = 0.481, *p* = 0.495	*F* = 0.018, *p* = 0.895	*F* = 2.141, *p* = 0.147
Decoy	***F* = 5.383**, *p* = 0.023	*F* = 0.877, *p* = 0.353	*F* = 3.605, *p* = 0.062	*F* = 0.121, *p* = 0.727
Included in the final model	age + decoy	date	year	—

*Note:* *F*‐ and *p* values are reported for the fixed effects. Significant *F*‐values are in bold. Individual identity was included in all models as a random factor and was retained in all final models.

**TABLE 4:** Results from the linear mixed models with the song traits as response variables and the control variables and the environmental variables as explanatory variables. Estimate ± standard error and *p* value are presented for fixed factors (significant values are in bold and marginally significant values are in italics) and variance is shown for random effects.

	Mean frequency	Frequency bandwidth	Complexity	Song length
Number of recordings (number of individuals)	65 (31)	69 (35)	68 (35)	69 (35)
Control variables				
Age (one‐year old)	**0.876** ± **0.288**, *p* = 0.004	—	—	—
Date	—	**0.406 ± 0.143**, *p* = 0.008	—	—
Decoy (without)	−0.323 ± 0.238, *p* = 0.179	—	—	—
Year (2021)	—	—	0.243 ± 0.431, *p* = 0.579	—
Year (2022)	—	—	0.640 ± 0. 460, *p* = 0.184	—
Environmental variables				
Shannon diversity index	0.117 ± 0.133, *p* = 0.386	−0.093 ± 0.127, *p* = 0.474	0.020 ± 0.125, *p* = 0.873	−0.017 ± 0.137, *p* = 0.901
Mean trunk circumference of sessile oaks	0.135 ± 0.116, *p* = 0.248	−0.133 ± 0.121, *p* = 0.284	−0.161 ± 0.136, *p* = 0.249	0.133 ± 0.125, *p* = 0.292
Health status	−0.162 ± 0.131, *p* = 0.220	*0.255 ± 0.129*, *p* = 0.063	0.169 ± 0.176, *p* = 0.347	−0.048 ± 0.133, *p* = 0.718
Canopy closure	**−0.303 ± 0.151**, *p* = 0.0499	0.129 ± 0.146, *p* = 0.388	−0. 075 ± 0.186, *p* = 0.687	0.178 ± 0.144, *p* = 0.222
Random terms				
ID	0	0.067	0.241	0
Residual	0.868	0.877	0.470	1.136

### 3.2 Relationships Between Song and Environmental Variables

**FIGURE 1:**
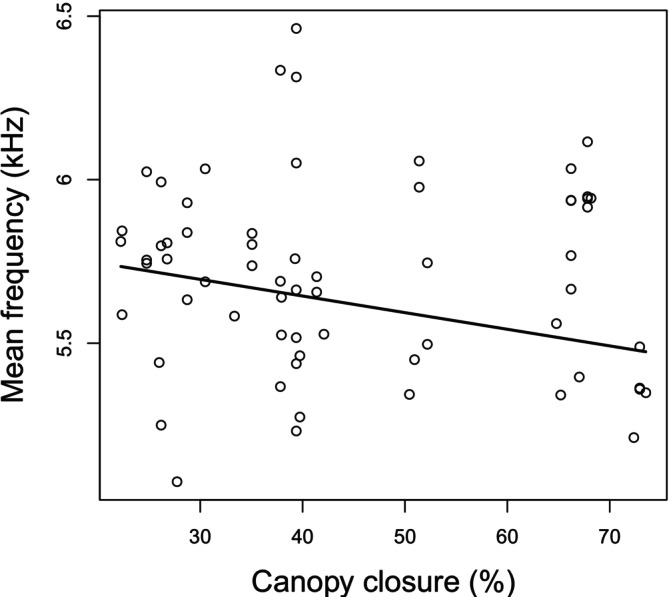
Relationship between mean frequency and canopy closure.

**FIGURE 2:**
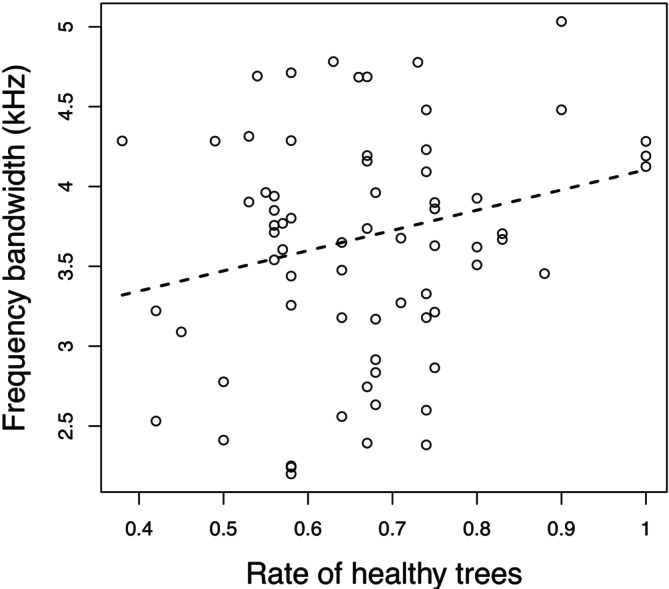
Relationship between frequency bandwidth and tree health status.

We found that mean frequency was significantly related to forest canopy closure (Table 4, Figure 1), as mean frequency decreased with increasing forest canopy closure. Moreover, we found a marginally significant positive trend between frequency bandwidth and the proportion of healthy sessile oak trees, as frequency bandwidth tended to increase with the proportion of healthy trees (Table 4, Figure 2). We did not find any significant relationship between the other song variables and any of the environmental variables (Table 4).

## 4 Discussion

In this field study, we found relationships between a song characteristic and habitat structure and quality. Specifically, the mean frequency of the songs was lower under closed canopy. This result may support the acoustic adaptation hypothesis or may be explained by the higher quality of males occupying better territories. Overall, our results highlight the role of habitat structure and quality in shaping bird song. Habitat‐dependent changes in bird song may affect sexual selection and reproductive success (Slabbekoorn and Ripmeester 2008; Laiolo 2010). Thus, understanding how habitat quality shapes birdsong and how songbirds adapt to diverse acoustic environments can provide key insights into the evolutionary dynamics of vocal communication in birds. Additionally, our results combined with previous findings indicate the potential of song as an indicator of habitat quality.

The significant negative relationship between mean frequency and forest canopy closure in collared flycatcher males could be attributed to environmental acoustics, as birds may adjust the frequency of their songs based on the acoustic properties of their environment (Nicholls and Goldizen 2006; Benedict and Warning 2017). Higher frequencies tend to be more easily absorbed by obstacles such as dense vegetation, like the tree canopy (Wiley and Richards 1982; Proppe et al. 2010). As the forest canopy closure increases, it might hinder the transmission of higher frequency sounds, leading to a negative correlation between mean frequency and canopy closure if birds modify their vocalisation adaptively, according to the acoustic adaptation hypothesis (Morton 1975). Moreover, dense vegetation, such as forest canopy, can produce background noise in wind. This noise was shown to be louder at higher frequencies (3000–8000 kHz; Morton 1970; Morton 1975), at the typical singing frequencies of the collared flycatcher. Birds may adjust the frequency of their songs to avoid being masked by this environmental noise, ensuring that their signals are more detectable to potential mates or rivals (Morton 1975). Although we did not make song recordings in strong wind, wind conditions can change rapidly, so singing lower in noisier territories with higher canopy closure (as the canopy closure variable reflected the development of the foliage) should be advantageous.

Alternatively, lower mean frequency may signal the quality of the singing bird through its relationship with body size (Ryan and Brenowitz 1985; Gil and Gahr 2002). Accordingly, there is evidence from various bird species that songs at lower frequencies are preferred by females (Pasteau et al. 2007; Halfwerk et al. 2011; Byers et al. 2015). High‐quality birds may obtain better territories with higher canopy closure (which can be associated with higher food resources, see above) and this can also result in an association between mean frequency and canopy closure. However, mean frequency and tarsus length (a common proxy of body size in birds) were not related in our sample, as revealed by an additional analysis (linear mixed model, estimate ± SE for tarsus: −0.003 ± 0.119, *t* = −0.028, *p* = 0.977) that makes the explanation that mean frequency reflects body size‐related quality less likely. Another possibility is that birds successfully occupying good territories adjust their song performance to signal their high‐quality resource, using lower frequency songs preferred by the females. However, it should be mentioned that the relationship between song frequency and female preference is not clear. In some species, high‐frequency songs are preferred (Byers 2006; Cardoso et al. 2007) and we have no evidence of association between pairing success and song frequency in the study species (Garamszegi, Møller, et al. 2004; Hegyi, Herényi, et al. 2010). High‐frequency songs can be honest signals of quality as high‐frequency vocalisations were found to be costly in various species (Araya‐Ajoy et al. 2009; Titze and Riede 2010). Further research and detailed behavioural observations could help uncover the underlying mechanisms driving this observed relationship between mean frequency and forest canopy closure. Additional studies are also needed to determine whether habitat‐dependent alterations are manifested on the among‐individual or within‐individual levels (implying plastic changes). We also should consider a mechanistic explanation for our results: higher frequencies under closed canopy became attenuated before reaching our recorder, biasing the results. However, we minimised this bias with our recording protocol and conducted additional analysis confirming the low effect of attenuation in the canopy on the recorded syllables (see Appendix A, Table A1).

There are multiple potential proximal mechanisms of the found change in mean frequency that could not be separated with our data. Males may sing other syllables with different frequency characteristics or the same syllables at lower or higher frequencies. However, we argue that not knowing the exact mechanism does not diminish the biological significance of our results. These mechanisms do not necessarily change repertoire size (Halfwerk et al. 2011) that frequently play a role in sexual selection (Gil and Gahr 2002; Beecher and Brenowitz 2005), but both can influence the preference of females (Halfwerk et al. 2011; Huet des Aunay et al. 2014). Furthermore, frequency‐dependent attenuation still affects songs of different frequency irrespective of their syllable composition. Importantly, regardless of the mechanism, the found mean frequency change of around 0.2 kHz can have biological meaning (note that the range of mean frequency was between 5 and 6.5 kHz in our data), as in great tits a difference of similar magnitude has consequences for reproductive success (Halfwerk et al. 2011).

Reflecting habitat characteristics in song can be relevant for mate choice if it conveys honest information from afar about habitat quality for the females (Hoi‐Leitner et al. 1995; Lampe and Espmark 2003). Thus, song can facilitate for females to find males with better territories. However, when birds adjust the acoustic properties of their songs to environments altered by humans, for example, noisy urban habitats, this could lead to detrimental effects on individual fitness (Laiolo 2010; Patankar et al. 2021; Hopkins et al. 2022). Nevertheless, bird song can be used as a habitat quality indicator also by forestry. According to our result, mean frequency can signal some aspect of deciduous forest quality, similarly as was suggested previously for song repertoire size in chipping sparrows ( *Spizella passerina* ) in open forests (Ortega et al. 2014) and song rate for multiple species in tropical forests with logging (Pillay et al. 2019).

The lack of further associations between other song traits and habitat characteristics can be attributed to the flexibility of the song of the collared flycatcher and the various factors that can shape it. Previously, we have demonstrated that among others, immune state, age, social environment and singing post height can influence song on either the among‐individual or within‐individual levels (Garamszegi, Møller, et al. 2004; Garamszegi et al. 2007; Jablonszky et al. 2022). Additionally, we know little about song crystallisation and song learning of the study species (Vaskuti et al. 2022). If they learn song on the wintering grounds, song can be shaped to a great extent by environmental effects there. Another explanation for our few significant findings can be the relative homogeneity of the woodland covering our study area. The trees of the dominant sessile oak are of similar age, reflected by their mean trunk circumference (see Table 2), but our other variables, such as canopy closure and Shannon diversity index, captured sufficient variation.

In sum, our results revealed habitat‐dependent variation in a song trait of the collared flycatcher. Male birds may adjust their songs to the acoustic transmission properties of their environment, and they may reflect the quality of their territories when singing. This habitat‐dependent adjustment can be important during mate choice. Furthermore, bird song can be used as a signal of habitat quality that could be useful in forest management.

## Author Contributions

**Katalin Krenhardt:** conceptualization (equal), formal analysis (equal), investigation (lead), writing – original draft (equal). **Mónika Jablonszky:** formal analysis (equal), investigation (equal), visualization (lead), writing – original draft (equal). **Karola Anna Barta:** investigation (equal), writing – review and editing (equal). **Miklós Laczi:** investigation (equal), writing – review and editing (equal). **Gergely Nagy:** investigation (equal), writing – review and editing (equal). **Sándor Zsebők:** data curation (equal), investigation (equal), writing – review and editing (equal). **László Zsolt Garamszegi:** conceptualization (equal), investigation (equal), writing – review and editing (equal).

## Conflicts of Interest

The authors declare no conflicts of interest.

## Data Availability

Data and code used to obtain the results were uploaded to Figshare: https://doi.org/10.6084/m9.figshare.29106830 (Krenhardt et al. 2025).

## Appendix A

### Text A1: Assessing the Potential Bias due to Frequency‐Dependent Attenuation

Higher frequencies usually became more attenuated than lower frequencies in densely vegetated ecosystems due to scattering (Wiley and Richards 1982). This phenomenon could have biased our estimation of mean frequency, especially for syllables sung at multiple frequencies in parallel. To assess this bias, we carried out additional analyses on an independent and larger dataset from our previous song recordings (recorded between 1999 and 2015 with the same protocol) where syllables were classified to syllable types across individuals. The sound analysis was otherwise similar to that described in the main text, and syllable types classification is detailed in our previous papers (Zsebők, Blázi, et al. 2018).

We selected the 10 most frequent syllable categories from the database and investigated the effect of singing post height on their mean frequency with linear mixed models containing also recording ID as a random factor. Significance for the fixed effect was calculated using a likelihood ratio test. We expected a negative relationship between mean frequency and singing post height if higher frequencies became more attenuated until they reached our microphone through the canopy. However, we did not find such a relationship (only a marginally negative one and a positive one contrary to our prediction) in the investigated syllables, regardless of their structure. These analyses, although they cannot completely prove that there are no such effects, suggest that the bias due to frequency‐dependent attenuation is not high in our data.

**TABLE A1:** Relationship between mean frequency and singing post height in the 10 most frequent syllable types. Recording ID was used as a random factor in all models. Estimate ± standard error (SE) and *p* value are presented for fixed factors (the significant value is in bold and a marginally significant value is in italics).

Syllable category	Number of observations (number of recordings)	Estimate ± SE for singing post height
772	5479 (166)	−0.223 ± 0.357, *p* = 0.536
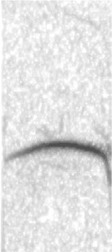
1289	1262 (72)	0.433 ± 0.540, *p* = 0.423
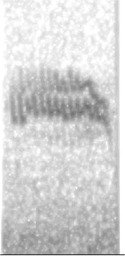
399	1455 (142)	**4.540 ± 1.563**, *p* = 0.004
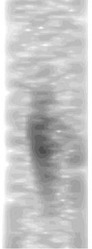
1915	739 (90)	0.325 ± 0.884, *p* = 0.713
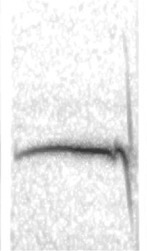
247	927 (105)	*−1.038 ± 0.588*, *p* = 0.078
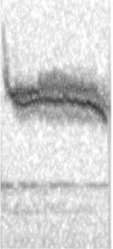
250	564 (62)	1.066 ± 0.999, *p* = 0.287
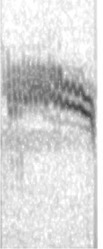
416	572 (50)	−0.611 ± 0.633, *p* = 0.335
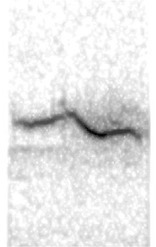
67	543 (87)	0.706 ± 0.939, *p* = 0.458
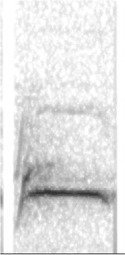
69	570 (66)	0.072 ± 0.946, *p* = 940
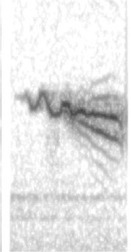
763	953 (68)	0.248 ± 0.266, *p* = 0.355
